# Alcohol Consumption in China Before and During COVID-19: Preliminary Results From an Online Retrospective Survey

**DOI:** 10.3389/fpsyt.2020.597826

**Published:** 2020-11-25

**Authors:** Yunfei Wang, Heli Lu, Maorong Hu, Shiyou Wu, Jianhua Chen, Ling Wang, Tao Luo, Zhenzhen Wu, Yueheng Liu, Jinsong Tang, Wei Chen, Qijian Deng, Yanhui Liao

**Affiliations:** ^1^Department of Psychiatry, The Second Xiangya Hospital, Central South University, Changsha, China; ^2^National Clinical Research Center of Mental Disorders, Changsha, China; ^3^Department of Psychosomatic Medicine, The Second Affiliated Hospital of Nanchang University, Nanchang, China; ^4^Department of Psychiatry, The First Affiliated Hospital of Nanchang University, Nanchang, China; ^5^The Third Affiliated Hospital of Guizhou Medical University, Qiannan, China; ^6^Shanghai Clinical Research Center for Mental Health, Shanghai Key Laboratory of Psychotic Disorders, Shanghai Mental Health Center, Shanghai Jiao Tong University School of Medicine, Shanghai, China; ^7^Florence Nightingale Faculty of Nursing, Midwifery & Palliative Care, King's College London, London, United Kingdom; ^8^Department of Social Medicine and Health Management, Xiangya School of Public Health, Central South University, Changsha, China; ^9^Jiangxi Mental Hospital, Nanchang, China; ^10^Department of Psychiatry, Sir Run Run Shaw Hospital, School of Medicine, Zhejiang University, Hangzhou, China; ^11^Key Laboratory of Medical Neurobiology of Zhejiang Province, Hangzhou, China; ^12^Addictions Department, Institute of Psychiatry, Psychology and Neuroscience, King's College London, London, United Kingdom

**Keywords:** alcohol consumption, heavy drinking, risky drinking, before and during COVID-19, retrospective survey

## Abstract

**Background:** Alcohol is an important aspect of Chinese culture, and alcohol use has been traditionally accepted in China. People with stress, anxiety, and depression may use more alcohol. More people reported symptoms of anxiety and depression during the outbreak of COVID-19. Thus, people may drink more alcohol during the outbreak of COVID-19 than before COVID-19.

**Methods:** An online retrospective survey was conducted on a total sample of 2,229 participants. Drinking behaviors before and during COVID-19, current risky drinking and hazardous drinking, and the association between high-risk drinking and mental health problems (depression, anxiety, and stress) were assessed *via* self-reported measures on the Alcohol Use Disorders Identification Test (AUDIT) and the 21-item Depression Anxiety Stress Scales (DASS−21).

**Results:** This study found that, compared with before COVID-19, alcohol consumption was slightly decreased during COVID-19 (from 3.5 drinks to 3.4 drinks, *p* = 0.035) in the overall sample. Most (78.7%) alcohol drinkers were males. Before and during COVID-19, males consumed more drinks per week (4.2 and 4.0 vs. 1.3 and 1.2 drinks), had a higher percentage of heavy drinking (8.1 and 7.7% vs. 4.4 and 2.7%), and more drinking days per week (2.1 and 2.1 vs. 1.0 and 0.9 days). Males also had more risky drinking (43.2 vs. 9.3%) and hazardous drinking (70.2 vs. 46.6%) than female counterparts. This study also found that high-risk drinking predicted anxiety in females.

**Conclusions:** This study suggests a slight reduction in alcohol consumption during COVID-19. However, hazardous drinking is common, especially among male alcohol drinkers. Males consumed more alcohol, had more risky and hazardous drinking than female counterparts both before and during COVID-19. Public health policy makers should pay more attention to developing effective, population-based strategies to prevent harmful alcohol consumption.

## Introduction

A 2018 global status report on alcohol and health by the World Health Organization (WHO) shows that the numbers of current drinkers (those who have consumed alcoholic beverages in the previous 12-month period) aged 15 and over reached more than two billion (accounting for 43% of the population) (1). There are only three WHO regions in which alcohol is consumed by more than half of the population, including the European region (EUR) (59.9% of current drinkers), the region of the Americas (AMR) (54.1%), and the Western Pacific region (WPR) (53.8%), and the increase in current drinkers in WPR is dominated by China (1). In 2016, alcohol use led to a large burden of disease and injury, causing 132.6 million disability-adjusted life years (DALYs, the total loss of healthy life years from onset to death), which represented 5.1% of all DALYs (1). The harmful use of alcohol is associated with more than 200 diseases and unintentional injury deaths (e.g., in 2016, approximately three million drinkers lost their lives due to harmful use of alcohol globally) (1, 2). The total alcohol consumption per capita remained at the level of 6.4 L (equivalent to 13.9 g of pure alcohol per day) globally over the period 2010–2016, but that was increased from 7.1 to 7.2 L in China (1). Consequently, the number of alcohol-attributable deaths also increased from 484,000 to 670,000 over the past decade (from 2007 to 2017) (3). The burden caused by alcohol use has become the third highest behavioral risk factor after inappropriate diet and smoking in China (4).

Alcohol use disorder (AUD), including alcohol abuse and dependence, is a chronic and relapsing disease, which is characterized by compulsive, heavy alcohol use and loss of control over alcohol intake (5). Globally, nearly 300 million adults (mainly males) suffer from AUD, and 2.6% of people aged 15 and over suffer from alcohol dependence (AD, the most severe form of AUD) (1). The current prevalence estimate of AD in China is about 2.2% (6). AD is characterized by craving, tolerance, and withdrawal (7) and is associated with physical and mental disease, causing impairments in overall health-related quality of life (HRQOL) (8). Data from 302 hospitals in Beijing, China, found that the number of hospitalized patients with alcoholic liver disease (ALD) increased from 1.68% in 2002 to 4.59% in 2013 (9).

There are several factors that can impact the levels and patterns of alcohol consumption, including biological, individual, and social factors (10). A meta-analysis of twin and adoption studies showed that about 50% of AUDs are heritable (11). The prevalence tends to be higher if alcohol is readily available, and people adopt a more permissive attitude toward heavy drinking (12, 13). Individuals with low socioeconomic status are at least twice as likely to die from their disorders and prolonged heavy alcohol use than their counterparts with high socioeconomic status (14). Negative emotions, such as stress, anxiety, and depression, are associated with alcohol use and may increase an individual's vulnerability to AUD (15–17). Alcohol is an important aspect of Chinese culture. Alcohol use has been traditionally accepted in China, especially during major social events (such as the spring festival and parties) (18). A survey of the general population in China found that males used alcohol more heavily and frequently than females (19).

An online survey of mental health problems caused by the COVID-19 outbreak and mass isolation found that, during the COVID-19 outbreak (*n* = 1,074), Chinese residents (mainly Hubei Province) reported a rate of 9.5% of harmful drinking and 1.6% of AD, and drinking problems were higher among people from Hubei (which had the most COVID-19 cases) than other provinces (20). Assessing the changes in Chinese people's drinking behavior before and during the outbreak of COVID-19 may provide evidence for public health policy making on preventing harmful alcohol consumption.

The primary objective of this online retrospective survey is to assess the impact of COVID-19 on alcohol drinking patterns among people from the general population of China by comparing alcohol drinking before and during the outbreak of COVID-19. Considering the association between alcohol use and anxiety and depression (21) and more people reporting symptoms of anxiety and depression during the outbreak of COVID-19 (20, 22), we hypothesize that, compared with before the outbreak of COVID-19, Chinese people may drink more alcohol during the outbreak of COVID-19. We also hypothesize that high-risk drinking would be associated with depression, anxiety, and stress.

## Methods

### Participants and Inclusion and Exclusion Criteria

In this cross-sectional, retrospective, anonymous online survey, participants were 18 years of age or older, Chinese literate, alcohol drinkers, able to access online services, had interest, and were willing to provide informed consent to participate in the study. An overview of participant eligibility criteria is given in Table 1.

**Table 1:** Inclusion and exclusion criteria of participants.

**Inclusion criteria**
1. 18 years of age or older
2. Alcohol drinker
3. Being able to access online services
4. Being able to read and write in Chinese
5. Expressing an interest in participant this study
6. Willing to provide informed consent to participate in the study
**Exclusion criteria**
1. Under 18 years of age
2. Unable to access online services
3. Unable to read and write in Chinese

### Recruitment

An Internet-based recruitment method was used in this cross-sectional survey study. We used Internet-based advertisements (such as websites and social media) to invite any adults to participate in an approximately 10- to 20-min online survey between May 7, 2020, and August 3, 2020. Advertisements contained a hyperlink that directed potential participants to the study's ethics approved consent form. After obtaining electronic informed consent, eligible participants were asked to complete a demographic questionnaire and measures of addictive behaviors (smoking behavior and the behavior of using social media and Internet gaming, reported elsewhere). Participants were required to answer all questions before submitting the survey but could quit the survey at any time. Thus, this survey had no missing data.

### Measures

#### Socio-Demographics

Socio-demographic information of all respondents included gender, age, years of completed education, employment status (employed, unemployed, student), marital status, and region (rural, urban).

#### Alcohol Drinking Behavior and Possible Alcohol Dependence

Alcohol consumption (number of alcohol units) before and during the outbreak of COVID-19 was measured by a Timeline Followback (TLFB) questionnaire (23), including questions about (1) average amount (mL) of alcohol intake per day, (2) average amount (mL) of alcohol intake per week, and (3) average alcohol drinking days per week (0–7).

The Alcohol Use Disorders Identification Test (AUDIT) (24, 25) is a widely used tool to assess hazardous drinking and AUDs. AUDIT is a comprehensive, 10-question screening tool. The total score ranges from 0 to 40, from low risk (0–7), increasing risk (8–15), higher risk (16–19), to possible dependence (20 or more). The AUDIT alcohol consumption questions AUDIT-C (Alcohol Use Disorders Identification Test for Consumption) (26, 27) is a modified version of the AUDIT and includes three items: (1) How often do you have a drink containing alcohol? (scores: never = 0, monthly or less = 1, 2-4 times per month = 2, 2-3 times per week = 3, 4+ times per week = 4); (2) How many units of alcohol do you drink on a typical day when you are drinking? (1–2 = 0, 3–4 = 1, 5–6 = 2, 7–9 = 3, 10+ = 4); (3). How often have you had 6 or more units if female or 8 or more if male on a single occasion in the last year? (Never = 0, Less than monthly = 1, Monthly = 2, Weekly = 3, Daily or almost daily = 4). The AUDIT-C is scored on a scale of 0–12. In male individuals, a score of ≥4 is considered positive, optimal for identifying hazardous drinking or active AUDs. In female individuals, a score of ≥3 is considered positive. Generally, the higher the score, the more likely it is that an individual's drinking is affecting his or her safety. The severity of risk drinking in AUDIT-C points are ranked as severe (8–12 points), high (6–7 points), moderate (4–5 for males and 3–5 for females), and low (0–3 for males and 0–2 for females).

#### Mental Health (Depression, Anxiety, and Stress)

The 21-item Depression Anxiety Stress Scale (DASS−21) (28) was used to identify signs of anxiety, depression, and stress. The DASS-21 has been translated and validated in Chinese (27, 29). It is a self-report instrument consisting of three subscales, and each of the three DASS-21 scales contains 7 items to assess depression, anxiety, and stress over the last week. The responses are given on a 4-point Likert scale, ranging from “I strongly disagree” (0) to “I totally agree” (3). Overall scores for the three constructs are calculated as the sum of scores for the relevant seven items multiplied by two. Ranges of scores correspond to levels of symptoms, ranging from “normal” to “extremely serious.” Scores for depression, anxiety, and stress are calculated by summing the scores for the relevant items, ranging from 0 to 21 scores. Recommended cutoff scores for conventional severity labels (normal, mild, moderate, severe, extremely severe) are as follows (multiplied by 2): depression: 0–9, 10–13, 14–20, 21–27, 28+; anxiety: 0–7, 8–9, 10–14, 15–19, 20+; stress: 0–14, 15–18, 19–25, 26–33, 34+.

### Definition

#### Alcohol Drinker

Defined as a person who drinks no <30 g alcohol (equal to 900 mL beer) per week.

#### Standard Drink

A standard drink is equal to 14.0 g (0.6 ounces, equal to 18 mL) of pure alcohol. Generally, this amount of pure alcohol is found in: 12 ounces (equal to 355 mL) of beer (5% alcohol content); 8 ounces (equal to 237 mL) of malt liquor (7% alcohol content); 5 ounces (equal to 148 mL) of wine (12% alcohol content); 1.5 ounces (equal to 44 mL) or a “shot” of 80-proof (40% alcohol content) distilled spirits or liquor (e.g., gin, rum, vodka, whiskey).

#### Heavy Drinker

If a man drinks more than 14 drinks per week or a woman drinks more than 7 drinks per week (30).

#### Hazardous Drinking or Active AUDs

Women who score 3 or higher in AUDIT-C recommended limits; men who score 4 or higher in AUDIT-C recommended limits (26).

### Investigation Methods/Data Collection

This online survey study was performed using a professional online survey service, Questionnaire Star (https://www.wjx.cn), released nationwide through social media (such as WeChat, Weibo, QQ, etc.).

### Quality Control

The computer IP address can be tracked to determine whether multiple entries were made from the same computer. However, considering that respondents may share the same Internet and same IP address with their families, this study did not limit the number of questionnaires from the same IP address. However, the survey allowed only one response per phone or computer.

### Data Analysis

All data was automatically collected by Questionnaire Star. A user-specified Excel file was downloaded from the database. Statistical analysis was performed using SPSS (IBM Corp. Released 2013. IBM SPSS Statistics for Windows, Version 22.0. Armonk, NY: IBM Corp.). Descriptive statistics, chi-square (χ^2^) tests, and independent sample *t-*tests were applied to measure differences in alcohol drinking behaviors between before and during the outbreak of COVID-19. Regression analysis was applied to explore whether high-risk drinking predicted depression, anxiety, and stress in males and females. Statistical significance was set for two-sided *p* < 0.05.

## Results

### Demographic Information

Demographic characteristics for the overall sample, males, and females are shown in Table 2. The total sample is 2,229 participants (78.7% males). Male participants were older, more employed, more married, and younger for first use of alcohol than their female counterparts. There were no differences between male and female participants in the rates of mental health problems with more than 30% individuals reporting depression, about 40% of people reporting anxiety, and more than 20% of them reporting stress during the last week of the survey.

**Table 2:** Demographic characteristics, overall and for males and females.

**Variables**	**Overall** **(*N* = 2,229)**	**Male** **(*n* = 1,755, 78.7%)**	**Female** **(*n* = 474, 21.3%)**	*p*-value^*^
Age (mean ± SD)	36.6 ± 9.92	37.5 ± 9.71	33.4 ± 10.03	<0.001
**Education (%)**				
High school or lower	11.2	11.0	11.8	0.616
College or higher	88.8	89.0	88.2	
**Employment (%)**				
Unemployed	3.3	2.8	5.1, 24	0.014
Employed	96.7	97.2	94.9	
**Marriage (%)**				
Married	71.7	75.6	57.4	<0.001
Unmarried	28.3	24.4	42.6	
**Living place (%)**				
Urban	96.6	96.4	97.3	0.367
Rural	3.4	3.6	2.7	
**Mental health problems (%)^#^**				
Depression	34.0	33.7	34.8	0.660
Anxiety	39.5	38.6	42.8	0.097
Stress	22.3	22.2	22.6	0.849
Age of first drinking	17.6 ± 4.98	17.3 ± 4.85	18.6 ± 5.31	<0.001

*Data are n (%) or mean (SD)*.

* *Significant difference, p < 0.05 (two sample t-tests for continuous variables and χ^2^ tests for categorical variables)*.

# *Mental health problems ranged from mild, moderate, severe, to extremely severe*.

### Drinking Behaviors Before and During COVID-19

Drinking behaviors before and during COVID-19 for the overall sample, males, and females are shown in Table 3. Compared to the time before COVID-19 and during COVID-19 in the overall sample, the average drinks (a standard drink is equal to 14.0 g or 0.6 ounces, = 18 mL, of pure alcohol) per week decreased from 3.5 drinks to 3.4 drinks, *p* = 0.035; the percentage of heavy drinking also insignificantly decreased from 7.4 to 6.7%, and the average of drinking days per week reduced from 1.9 to 1.8 days.

**Table 3:** Drinking behaviors before and during COVID-19, overall and for males and females.

	**Before COVID-19**	**During COVID-19**	*p*-value^*^
**Drinks per week (mean ± SD)**			
Overall	3.5 ± 6.29	3.4 ± 6.18	0.035
Male	4.2 ± 6.83	4.0 ± 6.71	0.070
Female	1.3 ± 2.59	1.2 ± 2.65	0.190
**Heavy drinker^#^ (%)**			
Overall	7.4, (164)	6.7, (149)	0.379
Male	8.1, (143)	7.7, (136)	0.662
Female	4.4, (21)	2.7, (13)	0.162
**Drinking days per week (mean ± SD)**			
Overall	1.9 ± 1.82	1.8 ± 1.90	0.030
Male	2.1 ± 1.83	2.1 ± 1.92	0.104
Female	1.0 ± 1.49	0.9 ± 1.55	0.104

# *Heavy drinker: if a man drinks more than 14 drinks per week or a woman drinks more than 7 drinks per week. A standard drink is equal to 14.0 g (0.6 ounces, equal to 18 mL) of pure alcohol*.

* *Significant difference, p < 0.05*.

### Gender Differences in Drinking Behaviors Before and During COVID-19

Gender differences in drinking behaviors before and during COVID-19 are shown in Table 4. Males reported about 4 drinks per week both before and during COVID-19, which was almost four times more than females; males also reported higher rates of heavy drinking (almost twice before and more than twice during COVID-19) and more drinking days per week (more than twice) than females both before and during COVID-19.

**Table 4:** Gender differences in drinking behaviors before and during COVID-19.

	**Males (*n* = 1,755)**	**Females (*n* = 474)**	*p*-value^*^
**Drinks per week (mean ± SD)**			
Before COVID-19	4.2 ± 6.83	1.3 ± 2.59	<0.001
During COVID-19	4.0 ± 6.71	1.19 ± 2.650	<0.001
**Heavy drinker^#^ (%)**			
Before COVID-19	8.1	4.4	<0.001
During COVID-19	7.7	2.7	<0.001
**Drinking days per week (mean ± SD)**			
Before COVID-19	2.1 ± 1.83	1.0 ± 1.49	<0.001
During COVID-19	2.1 ± 1.92	0.9 ± 1.55	<0.001

# *Heavy drinker: if a man drinks more than 14 drinks per week or a woman drinks more than 7 drinks per week*.

* *Significant difference, p < 0.05*.

### Risky and Hazardous Drinking

Severity of drinking risks by AUDIT-C overall and for males and females are shown in Table 5. The overall high-risk drinking was 36% with 43.2% of males and 9.3% of females. Among high-risk drinkers, half of males and one-third of females may have AD. More than 70% of males and almost half of females (46.6%) reported hazardous drinking.

**Table 5:** Severity of drinking risks by AUDIT-C, overall and for males and females.

**Drinking risks**	**Overall** **(*n* = 2,229) % (*n*)**	**Males** **(*n* = 1,755) % (*n*)**	**Females** **(*n* = 474) % (*n*)**	*p*-value^*^
**High-risk drinking (AUDIT-C >5)**	36.0 (802)	43.2 (758)	9.3 (44)	<0.001
Severe, possible	19.3 (428)	23.4 (411)	3.6 (17)	
dependence				
High	16.8 (374)	19.8 (347)	5.7 (27)	
**Low-risk drinking (AUDIT-C 0-5)**	64.0 (1,427)	56.8 (997)	90.7 (430)	<0.001
Moderate	–	27 (474)	37.3 (177)	
Low	–	29.8 (523)	53.4 (253)	
Hazardous drinking		70.2 (1,232)	46.6 (221)	<0.001

*AUDIT-C, alcohol use disorders identification test for consumption*.

*Hazardous drinking: women who score 3 or higher in AUDIT-C recommended limits; men who score 4 or higher in AUDIT-C recommended limits*.

* *Significant difference, p < 0.05*.

### High-Risk Drinking Predicting Depression, Anxiety, and Stress

A regression model of high-risk drinking predicting depression, anxiety, and stress is shown in Table 6. Regression analysis found no association between high-risk drinking and mental health problems of depression, anxiety, and stress in males. However, high-risk drinking predicted anxiety in females.

**Table 6:** Regression model of high-risk drinking predicting depression, anxiety, and stress.

**Variable^#^**	**OR**	**CI**	*p*-value^*^
**Male**			
Depression	0.99	0.73–1.34	0.962
Anxiety	1.09	0.82–1.45	0.566
Stress	0.90	0.68–1.20	0.479
**Female**			
Depression	1.01	0.43–2.40	0.976
Anxiety	2.62	1.14–6.01	0.023
Stress	0.56	0.24–1.35	0.198

# *Normal as reference; OR, odds ratios; CI, 95% confidence intervals*.

* *Significant difference, p < 0.05*.

## Discussion

This online retrospective survey found that, compared with before COVID-19, alcohol consumption was slightly decreased during COVID-19. Most (78.7%) alcohol drinkers were males. They consumed more alcohol and had more risky and hazardous drinking than female counterparts both before and during COVID-19. This study also found that high-risk drinking predicted anxiety in females.

Compared with the time of before COVID-19, the average drinks per week, percentage of heavy drinking, and average drinking days per week all showed a reduction during COVID-19. A similar online survey of mental health problems caused by the COVID-19 outbreak and mass isolation during the earlier COVID-19 outbreak reported that Chinese residents from Hubei Province reported a higher rate of hazardous (33.5%) and harmful alcohol use (11.1%) than those from other provinces (21.5 and 1.9%, respectively); both rates are lower than the rates in the current study (20). The high rate of problematic drinking among Hubei residents may be due to distress experienced as a result of the pandemic. The result in our current study of lowered levels of consumption may be due to the decreased physical and financial availability of alcohol as well as the reduction of social interactions, such as celebrations and birthday parties (31). Our result shows that, in China, overall, alcohol consumption during the epidemic was lower than that before the epidemic. Although the Chinese government did not release a stricter alcohol control policy, due to strict national isolation and traffic control measures during the epidemic (32), the decline in alcohol availability is likely to be the main reason for the reduction of alcohol use. Interestingly, a study in Italy showed that, during the COVID-19 lockdown period, the craving level of inpatients was lower than real-life samples of outpatients with substance use disorders, and this may be related to the lack of availability of the substance and has relevant therapeutic implications for addicted patients (33). Further monitoring of alcohol drinking patterns during the COVID-19 pandemic would be necessary to better understand the effects of COVID-19 on alcohol consumption and to develop better comprehensive alcohol control policies.

The current study found gender differences in drinking behaviors before and during COVID-19. Males reported about 4 drinks per week both before and during COVID-19, which was almost four times more than females; males also reported higher rates of heavy drinking (almost twice before and more than twice during COVID-19) and more drinking days per week (more than twice) than females both before and during COVID-19. These differences are consistent with the results of previous studies in China and abroad, i.e., men drink more than women (19, 34, 35).

We also found more than one-third (36%) of high-risk drinking with 43.2% in males and 9.3% in females. Among high-risk drinkers, half of males and one-third of females may have AD. In addition, more than 70% of males and almost half of females (46.6%) reported hazardous drinking. These gender differences may partly be due to the different roles of men and women in society and family (36). Men reported drinking more when exposed to pleasant emotions and social pressure and drinking more, more frequently, and with a greater risk of harmful drinking than women (1, 37, 38). Women reported drinking more when experiencing family interpersonal problems, the death of someone close, and negative emotions (36, 39). This study also found high-risk drinking predicted anxiety in females but not in males. There is increasing concern about the increase in alcohol consumption and alcohol-related harms during the COVID-19 pandemic, such as AUD, intimate partner violence, harm to children, suicide, and non-communicable diseases (40). For example, interpersonal violence (especially intimate partner violence) may cause physical and mental harm to women (or men) during quarantine, which may be related to harmful alcohol consumption (41).

### Limitations

This study has some limitations that need to be considered. First, this online survey study only recruited participants online (many through social media, such as WeChat, QQ). As a result, more than 90% of the participants were from urban areas, and almost 90% of the participants had a college or higher degree; thus, the sample is not representative of the general adult population in China. Another limitation is that the measurements were all self-reported, which may result in bias of reported alcohol consumption; individuals who had drinking problems may be underestimated in this study. Furthermore, this is a retrospective survey, and alcohol consumption reported before COVID-19 may not be accurate. Further prospective cohort studies are needed to explore how social events affect alcohol drinking patterns and hazardous drinking as well as measures to prevent harmful alcohol use.

## Conclusions

The present study provides an insight into the impact of the COVID-19 pandemic on alcohol use patterns. This study suggests a slight reduction in alcohol consumption during COVID-19. However, hazardous drinking is common, especially among male alcohol drinkers. Males consumed more alcohol and had more risky and hazardous drinking than female counterparts both before and during COVID-19. Public health policy makers should pay more attention to developing effective, population-based strategies to prevent harmful alcohol consumption.

## Data Availability Statement

All data in the current study was stored in the PI's affiliation, and is available from the corresponding authors on reasonable request and with completion of data user agreement.

## Ethics Statement

The study protocol has been approved by The Ethics Committee of Sir Run Run Shaw hospital, an affiliate of Zhejiang University, Medical College (No. 20200505-33). Informed consent was communicated and obtained from each participant prior to participation. Participants were informed with the purpose, assessments, potential risks and benefits of the study before survey. All of the obtained data have no personal information, none information from the data can be linked back to the participants.

## Author Contributions

YW, QD, and YLiao conceived the study. YW, QD, and YLiao did the literature review, statistical analyses, and drafted the report. YW, QD, HL MH, SW, JC, LW, TL, ZW, and YLiu collected the data. YLiao and YW took the lead in writing the manuscript. JT and WC interpreted the data and commented on the manuscript. All authors contributed to the article and approved the submitted version.

## Conflict of Interest

The authors declare that the research was conducted in the absence of any commercial or financial relationships that could be construed as a potential conflict of interest.

## Abbreviations

WHO: World Health Organization
WPR: Western Pacific Region
DALYs: disability-adjusted life years
AUD: alcohol use disorder
AD: alcohol dependence
ALD: alcoholic liver disease
TLFB: Timeline Followback
AUDIT: Alcohol Use Disorders Identification Test
AUDIT-C: Alcohol Use Disorders Identification Test for Consumption
DASS−21: the 21-item Depression Anxiety Stress Scales.
